# Triple-Negative, HER2/Neu, and Ki67 Markers in Breast Cancer Patients Undergoing Standard of Care Treatment in India: Real-World Evidence on Tumor Aggressiveness and Survival Outcomes

**DOI:** 10.7759/cureus.78798

**Published:** 2025-02-09

**Authors:** Pravesh Dhiman, CN Patil, Sneha Konimeni, Meenakshi Meenu

**Affiliations:** 1 Medical Oncology, All India Institute of Medical Sciences Bilaspur, Bilaspur, IND; 2 Medical Oncology, Aster Cauvery Medical Institute (CMI) Hospital, Bengaluru, IND; 3 Pharmacology, All India Institute of Medical Sciences Bilaspur, Bilaspur, IND

**Keywords:** breast cancer, her2/neu, hormone receptor, ki67, triple-negative breast cancer

## Abstract

Introduction: Breast cancer is among the most prevalent cancers in women globally, with patients’ survival adversely impacted by Ki67 expression and triple-negative phenotypes. In this study, we examined the relationship between HER2/neu, triple-negative, and Ki67 phenotypes and tumor aggressiveness along with the survival of breast cancer patients from India.

Materials and methods: A retrospective cohort study was performed using hospital-based data from a tertiary care center spanning January 2013 to August 2023. The study included breast cancer patients who received neoadjuvant chemotherapy based on preoperative assessment and/or adjuvant chemotherapy following postoperative evaluation. Patients with other primary cancers or those treated with investigational drugs were excluded. Data on variables such as age, parity, menopause, cancer stage and grade, estrogen receptor (ER), progesterone receptor (PR), HER2/neu, Ki67 score, and the use of biologicals, hormones, and chemoradiotherapy were analyzed using correlation and regression tests to identify factors associated with aggressive tumor behavior. Kaplan-Meier survival model and Cox-proportional hazard test were applied.

Results: A total of 389 breast cancer patients with a mean age of 54.3 years met the eligibility criteria and were included in the analysis. A higher prevalence of hormone receptor positivity was observed among Indian patients than in Western countries. Younger, premenopausal women were more likely to present with high-grade tumors and high Ki67 scores, poorer overall survival, and the need for chemoradiotherapy. On the other hand, nulliparous patients mostly had triple-negative tumors with high Ki67 scores. Aggressive tumor behavior was linked to HER2/neu positivity, ER and PR negativity, and the requirement for both neoadjuvant and adjuvant chemotherapy. At the same time, premenopausal patients more frequently were candidates for radiation therapy. HER2/neu expression demonstrated a moderate negative correlation with co-expression of hormone receptors (ER and PR). Cancer grade was associated with Ki67 levels, nulliparity, triple-negative status, and hormone receptor expressions (ER and PR). Median survival was not reached for the study cohort.

Conclusion: Tumor aggressiveness was associated with a high Ki67 score, HER2/neu positivity, and the absence of hormone receptor expression in patients.

## Introduction

Breast cancer is among the most prevalent cancers in women globally [1]. Worldwide, as of 2020, 2.3 million breast cancer patients were diagnosed globally across both sexes, making it one of the most prevalent cancers. Currently, breast cancer accounts for one in eight cancer diagnoses worldwide. It is estimated that 685,000 women succumbed to the disease, contributing to 16% of all cancer-related deaths among women globally [2]. Breast cancer is the most common malignancy and the leading cause of cancer-related deaths among women in India. It constitutes 13.5% of all new cancer cases among Indian women and accounts for 10.6% of cancer-related deaths in this group [3]. Its pathogenesis is highly heterogeneous, resulting from a complex genetic, hereditary, and environmental interaction. Key risk factors include early onset of menstruation, late menopause, advanced maternal age at first pregnancy, nulliparity, inadequate breastfeeding, use of oral contraceptives, and hormone replacement therapy. Modifiable lifestyle factors, such as obesity, physical inactivity, and alcohol consumption, also contribute to risk [4]. Molecular characteristics, such as activation/mutation of HER2/neu, estrogen receptor (ER), progesterone receptor (PR), breast cancer 1/2 (BRCA1/2) gene, and programmed death-ligand 1 (PD-L1), play a crucial role in guiding the management of cancer [5]. Ki67, a nuclear antigen expressed during active cell cycles and indicative of cancer cell proliferation, is an emerging biomarker with diagnostic, prognostic, and predictive value. Changes in Ki67 scores provide insights into the efficacy of neoadjuvant therapy during the preoperative period and help estimate recurrence-free survival postoperatively [6]. Ki67 expression is categorized into low-risk (0-15%) and high-risk (>15%) based on the percentage of positively stained tumor cells [7]. Low-risk Ki67 is nonlinearly associated with survival, while high-risk Ki67 shows a linear relationship with disease-free and overall survival in breast cancer patients [8].

Histological classifications, including mucinous, medullary, and tubular carcinomas, can also influence prognosis. Tumors smaller than 5 mm generally have favorable outcomes regardless of histology; however, molecular features such as triple-negative or HER2-positive status negatively impact prognosis compared to ER-positive tumors of similar histology and stage [9-11]. Breast cancer is managed with a combination of surgery, pre- and/or postoperative chemotherapy, and radiotherapy [12]. Neoadjuvant chemotherapy, often incorporating targeted agents or immunotherapies, is particularly relevant for HER2-positive and triple-negative cancers, alongside risk-adapted postoperative strategies [12]. Hormonal therapy is typically used for early-stage breast cancer, while advanced-stage cases are often managed with palliative care [12]. This study analyzed the correlation between patient and tumor characteristics, age, parity, menopausal status, ER-PR expression, HER2/neu overexpression or amplification, and histological types and their impact on tumor behavior, treatment response, and overall survival.

## Materials and methods

A retrospective cohort study was conducted using hospital-based data from a tertiary care center in Bengaluru, India, between January 2013 and August 2023. The study did not require ethics approval as per institutional policy. The principles outlined in the Declaration of Helsinki had been followed.

Objective

The objective was to find the association of patient characteristics, such as age, parity, and menstrual status, and tumor characteristics, such as Ki67 score, triple-negative status, ER, PR, and HER2/neu expressions, with tumor aggressiveness and overall survival of breast cancer patients.

Eligibility criteria

Patients were eligible if they had a confirmed diagnosis of breast cancer staged according to the American Joint Committee on Cancer (AJCC) - Tumor, Node, Metastasis (TNM) system (NCCN Guidelines) [12]. Treatment included neoadjuvant chemotherapy based on preoperative assessment and/or adjuvant chemotherapy based on postoperative evaluation by the treating oncologist. Exclusion criteria included a history of any other primary malignancy before the breast cancer diagnosis or being treated with an investigational drug.

Data extraction

Data for those patients who satisfied eligibility criteria were recorded for this study. Age, parity, premenopausal/postmenopausal status, and other demographic details were recorded for all patients. The patients were diagnosed based on clinical features, confirmed by biopsy, and examined for histopathological and immunohistochemistry characteristics. Tumor size was also noted for all patients. The patients' molecular characteristics were recorded, including ER, PR, HER2/neu expressions, and Ki67 score for proliferation status. Bone and other metastases were evaluated based on clinical features and radiological examinations. A summation of these features established the stage and grade for all patients. These patients were treated using a combination of surgery, neoadjuvant and/or adjuvant chemotherapy, and radiotherapy. Based on the stage and grade, the patients either underwent breast-conserving surgery or modified radical mastectomy. A surgical oncologist, a breast surgeon, or a general surgeon did the operations. Data on age, parity, premenopausal/postmenopausal state, stage, grade, ER, PR, HER2/neu expressions, triple-negative cancer, Ki67 score for malignant cell proliferation, use of biologicals, hormonal and radiation therapy, type of surgery and surgeon, and administration of neoadjuvant and/or adjuvant therapies were recorded. After recording, data was anonymized by removing all direct identifiers, including names, addresses, phone numbers, and unique health identification numbers, before analysis.

Data analysis

The data had been presented as N (%) and mean ± SD. Pearson’s chi-square test with no continuity correction and correlation and regression analyses were done to identify the factors, including patient and tumor characteristics such as Ki67 score, ER-PR, HER2/neu, and triple-negative status, significantly influencing tumor behavior. Continuous variables were assessed using Pearson’s correlation test, while tetrachoric and polychoric correlation tests were used to find associations between binary and ordinal variables. The Cox proportional hazard model was used to explore whether the variables showed significant differences with respect to the patient’s survival. The hazard ratio (HR) indicates the relative risk of an event (e.g., recurrence or death), with values greater than 1 suggesting an increased risk and values below 1 indicating a reduced risk. The p-values assessed the statistical significance, with a common threshold of less than 0.05 to denote significance for all data analyses. Missing data was handled using a data deletion approach without making assumptions about the missing values. The data replacement strategy was not applied to the patients with significant clinicopathological characteristics.

## Results

A total of 389 breast cancer patients met the eligibility criteria and were included in the analysis. The frequency distribution of age for the cohort is detailed in Table 1.

**Table 1:** Frequency distribution of age for the study cohort Data presented as number (percentage) (N (%))

Age (years)	N (%)
<45	83 (21.3)
45-55	128 (32.9)
>55	178 (45.8)

The mean age was 54.3 years, with 256 patients (66%) being postmenopausal and 14 patients (3.6%) identifying as nulliparous. Among the cohort, 241 patients (62%) were ER-positive, and 217 (56%) were PR-positive. Molecular characteristics revealed two cases (0.5%) of ER-/PR+, 24 cases (6.2%) of ER+/PR-, 102 cases (26%) of HER2/neu+, 81 cases (21%) of triple-negative breast cancer, and 122 cases (31.4%) of Ki67 positivity. Only 26 events (6.7%) were recorded across the entire group. A total of 266 patients (68.4%) underwent modified radical mastectomy and did not require radiation therapy. Neoadjuvant chemotherapy was administered to 62 patients (16%), adjuvant chemotherapy to 276 patients (71%), and radiotherapy to 241 patients (62%). An equal number of patients (241; 62%) also received hormonal therapy. The characteristics of the Ki67 score, triple-negative biomarker, HER2/neu expression, and other clinicopathological variables of the study cohort were summarized in Tables 2-5, respectively.

**Table 2:** Association of demographic and tumor characteristics with Ki67 expression in breast cancer patients in India Values are presented as numbers (percentages) (N (%)). The chi-square test was applied for test statistics; p<0.05 was taken for statistical significance. Stages 1, 2, 3, and 4 are per the AJCC TNM staging system. ER.PR: estrogen and progesterone receptors co-expression, AJCC TNM: American Joint Committee on Cancer Tumor, Node, and Metastasis [12]

Study variables	Ki67 score		Test statistic	p-value
Age (years)	1-15%	>15%	χ2 ≈ 2.48	0.29
<45	23 (9.8)	30 (12.7)		
45-55	27 (11.4)	36 (15.3)		
>55	64 (27.1)	56 (23.7)		
Tumor stage			χ2 ≈ 0.0	1.0
Stage 1 & 2	74 (31.2)	80 (33.9)		
Stage 3 & 4	40 (16.9)	42 (17.8)		
Grade			χ2 ≈ 0.161	0.923
1	6 (2.7)	7 (3.1)		
2	71 (31.8)	74 (33.2)		
3	30 (13.5)	35 (15.7)		
Triple negative			χ2 ≈ 1.58	0.208
Negative	95 (40.6)	91 (38.9)		
Positive	19 (8.1)	29 (12.4)		
ER.PR receptors co-expression			χ2 ≈ 0.99	0.320
Negative	36 (16.4)	46 (21.0)		
Positive	71 (32.4)	66 (30.1)		
HER2/neu			χ2 ≈ 0.33	0.568
Negative	81 (34.8)	91 (39.1)		
Positive	32 (13.7)	29 (12.5)		

**Table 3:** Association of demographic, reproductive, and tumor characteristics in triple-negative breast cancer patients Values are presented as numbers (percentages) (N (%)). The chi-square test was applied for test statistics; *p<0.05 was taken for statistical significance. Stages 1, 2, 3, and 4 are per the AJCC TNM staging system. AJCC TNM: American Joint Committee on Cancer Tumor, Node, and Metastasis [12]

Study variables	Triple negative		Test statistics	p-value
Age (years)	No	Yes		
<45	60 (15.6)	22 (5.7)	χ2 ≈ 4.73	0.094
45-55	95 (24.7)	30 (7.8)		
>55	149 (38.7)	29 (7.5)		
Tumor stage				
Stage 1 & 2	203 (52.7)	57 (14.8)	χ2 ≈ 0.23	0.631
Stage 3 & 4	101 (26.2)	24 (6.2)		
Nulliparous				
No	295 (76.8)	75 (19.5)	χ2 ≈ 2.89	0.089
Yes	8 (2.1)	6 (1.6)		
Premenopausal				
No	205 (53.7)	47 (12.3)	χ2 ≈ 2.46	0.117
Yes	96 (25.1)	34 (8.9)		
Grade				
1	32 (8.8)	1 (0.27)	χ2 ≈ 34.94	<0.0001*
2	196 (53.8)	36 (9.9)		
3	58 (15.9)	41 (11.3)		

**Table 4:** Association of demographic, reproductive, and tumor characteristics with HER2/neu expression in breast cancer patients Values are presented as numbers (percentages) (N (%)). The chi-square test was applied for test statistics; *p<0.05 was taken for statistical significance. Stages 1, 2, 3, and 4 are per the AJCC TNM staging system. ER.PR: estrogen and progesterone receptors co-expression, AJCC TNM: American Joint Committee on Cancer Tumor, Node, and Metastasis [12]

Study variables	HER2/neu expression		Test statistics	p-value
Age (years)	Negative	Positive	χ2 ≈ 2.93	0.231
<45	63 (16.5)	18 (4.7)		
45-55	85 (22.2)	40 (10.5)		
>55	133 (34.7)	44 (11.5)		
Tumor stage			χ2 ≈ 3.34	0.341
Stage 1 & 2	193 (51)	66 (17)		
Stage 3 & 4	88 (23)	36 (9)		
Grade			χ2 ≈ 13.84	0.0009*
1	31 (8.5)	2 (0.55)		
2	156 (43.1)	75 (20.7)		
3	79 (21.8)	19 (5.25)		
Premenopausal			χ2 ≈ 1.197	0.274
No	180 (47.4)	71 (18.7)		
Yes	100 (26.3)	29 (7.6)		
Nulliparous			χ2 ≈ 1.85	0.174
No	268 (70.1)	100 (26.2)		
Yes	13 (3.4)	1 (0.3)		
ER.PR receptors co-expression			χ2 ≈ 26.7	<0.0001*
No	84 (23.5)	58 (16.3)		
Yes	181 (50.7)	34 (9.5)		

**Table 5:** Association of hormonal receptor expression with tumor stage in breast cancer patients Values are presented as numbers (percentages) (N (%)). The chi-square test was applied for test statistics; p<0.05 was taken for statistical significance. Stages 1, 2, 3, and 4 are per the AJCC TNM staging system. ER: estrogen receptor, PR: progesterone receptor, ER.PR: estrogen and progesterone receptors co-expression, AJCC TNM: American Joint Committee on Cancer Tumor, Node, and Metastasis [12]

Hormonal profile	Tumor stage		Test statistics	p-value
ER	Stage 1 & 2	Stage 3 & 4	χ2 ≈ 0.58	0.45
Negative	103 (26)	43 (11)		
Positive	161 (42)	80 (21)		
PR			χ2 ≈ 0.0028	0.96
Negative	116 (30)	54 (14)		
Positive	148 (38)	69 (18)		
ER.PR receptors co-expression			χ2 ≈ 0.000	0.99
Negative	116 (30)	54 (14)		
Positive	148 (38)	69 (18)		

**Figure 1:**
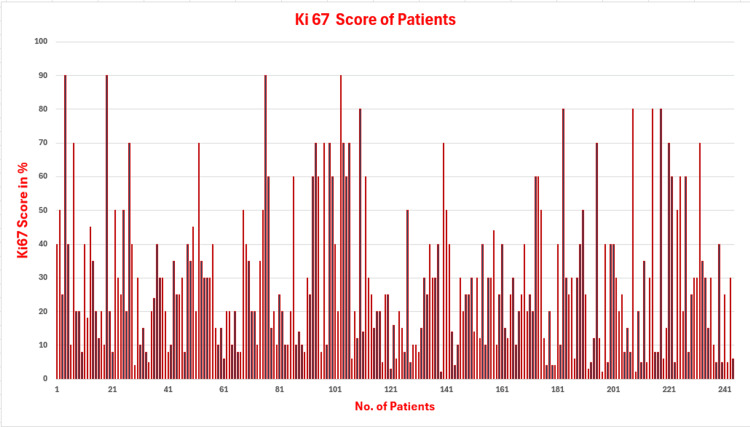
Frequency distribution of Ki67 score of breast cancer patients

Figure 1 shows the frequency distribution of Ki67 in this patient cohort. Missing data was handled using the data deletion approach for respective variables only, and the rest of the available data were included in the analysis (Appendices).

Survival characteristics

The HR of 1.00 indicated no association between age and survival in this cohort, as confirmed by a non-significant p-value of 0.72. Nulliparous women had a slightly elevated HR of 1.23. High-grade tumors had an HR of 1.39, implying a moderate increase in risk. This trend aligned with the known aggressive nature of higher-grade tumors. ER positivity was associated with an HR of 0.55, suggesting a lower risk than ER-negative patients. This aligns with the general understanding that ER-positive tumors often had a more favorable prognosis. PR expression showed an HR of 0.98, close to 1, with a non-significant p-value of 0.94, indicating that it did not significantly affect survival in this cohort. HER2/neu positivity showed an HR of 0.89, suggesting a slight reduction in risk due to the use of drugs targeting HER2/neu-positive tumors; however, the p-value (0.78) indicated no significant impact on survival outcomes consistent with mixed findings in other studies. The low Ki67 score, a marker of cellular proliferation, showed an HR of 0.86, suggesting a slight reduction in risk, but this was not statistically significant (p=0.74). Triple-negative tumors had an increased HR of 1.64, implying a higher risk with the known aggressive behavior of triple-negative breast cancer. Patients who received chemotherapy exhibited an HR of 4.10, indicating a strong association with increased risk, nearing statistical significance (p=0.06). This might reflect the aggressive cancers in patients selected for chemotherapy, often reserved for higher-risk cases, rather than a direct effect of chemotherapy. Neoadjuvant chemotherapy showed an HR of 1.20, which suggested a slight increase in risk. Neoadjuvant chemotherapy was typically used in more aggressive cases, which might explain the trend toward increased risk. Radiotherapy had an HR of 1.77, suggesting a higher risk, potentially reflecting its use in more advanced cases. Hormonal therapy showed an HR of 0.88, indicating a slight protective effect. Hormonal therapy generally benefited both ER/PR-positive patients, potentially reducing the risk of recurrence or progression. Most findings were not statistically significant at p<0.05 for this patient cohort.

Overall, traditional indicators of aggressive breast cancer, such as high-grade and triple-negative status, were associated with increased risk. Chemotherapy and radiotherapy were also linked to higher HRs, likely due to their use in advanced or high-risk cases. In contrast, hormonal therapy and ER positivity appeared to offer protective benefits, though most findings were not statistically significant for this patient cohort.

Correlation of tumor characteristics

Age was categorized into a binary variable, 1 for less than 45 years and 2 for more than 45 years, to look for any correlation with other covariates. We found statistically significant correlations among tumor characteristics (p<0.05). Age had a weak negative correlation with grade (point biserial correlation coefficient or rpc=0.20, p=0.0002), chemotherapy (rpc=0.22, p=0.0007), radiotherapy (rpc=0.23, p=0.0009), and with modified radical mastectomy (rpc=0.20, p=0.0004). Premenopausal patients were more likely to require radiotherapy (tetrachoric correlation coefficient or rtc=0.37, p=0.0001). Nulliparity had a weak correlation with triple-negative cancer (rtc=0.28, p=0.09) and a moderate correlation with high-risk Ki67 score (rtc=0.44, p=0.007). HER2/neu expression had a moderate negative correlation with hormone receptors (ER and PR) expression (rtc=-0.36, p=0.0003). The grade of cancer was correlated with Ki67 (polychoric correlation coefficient or rpe=0.23), nulliparity (rpe=0.22), triple-negative cancer (rpe=0.47), and ER-PR expression (rpe=-0.36). Significant findings on correlation coefficient and p-value for ER-PR+, HER2/neu, and Ki67 status with breast conservative procedure, chemotherapy, and radiotherapy, respectively, are presented in Table 6.

**Table 6:** Correlation analysis on ER+PR+, HER2/neu, and Ki67 with breast conservative procedure, chemotherapy, and radiotherapy, respectively *p<0.05 for statistical significance, Pearson's correlation coefficient test for data analysis ER+PR+: estrogen and progesterone receptor positive

Study variable	Treatment	Correlation coefficient (p-value)
ER+PR+	Breast conservative procedure	0.31 (0.004)*
HER2/neu	Chemotherapy	-0.4 (0.0005)*
Ki67	Radiotherapy	0.23 (0.007)*

Kaplan-Meier survival analysis

**Figure 2:**
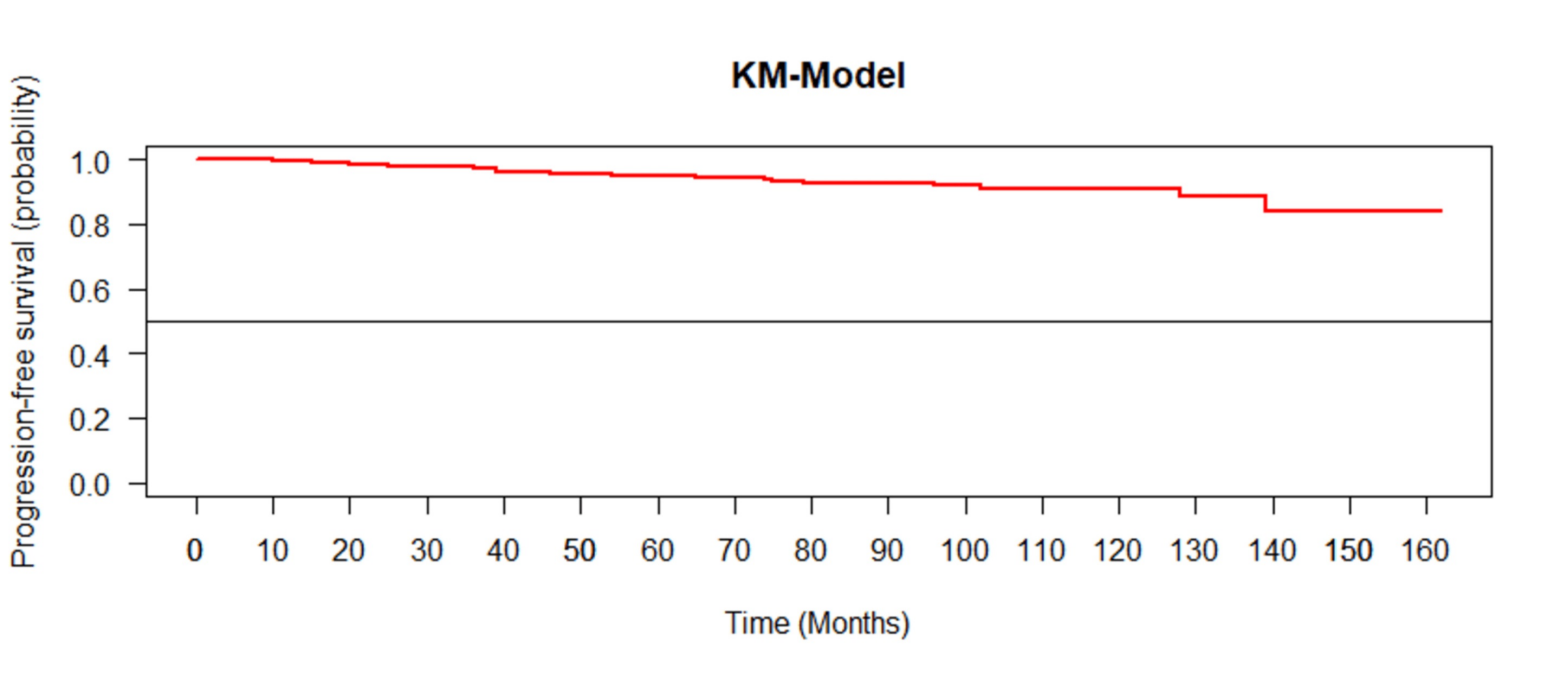
Kaplan-Meier survival curve (median survival was not reached for the study cohort)

The flat slope in the Kaplan-Meier survival curve suggested a low event rate and better survival prognosis (Figure 2).

Median survival was not reached for the study cohort. The Cox proportional HR was insignificant for any of the study variables, as summarized in Table 7.

**Table 7:** Cox proportional hazard analysis for the study variables Statistical test: Cox proportional hazard model, p<0.05 for statistical significance CI: confidence interval, ER: estrogen receptor, PR: progesterone receptor

Study variable	HR (95% CI)	p-value
Age	1.00 (0.971, 1.044)	0.72
Nulliparity	1.23 (0.166, 9.136)	0.84
Premenopausal state	0.59 (0.236, 1.462)	0.25
Grade	1.39 (0.690, 2.767)	0.36
ER	0.55 (0.255, 1.197)	0.13
PR	0.98 (0.4475, 2.105)	0.94
HER2/neu negative	0.89 (0.372, 2.11)	0.78
Ki67 low score	0.86 (0.352, 2.102)	0.74
Triple negative	1.64 (0.712, 3.774)	0.25
Chemotherapy	4.10 (0.967, 17.4)	0.06
Neoadjuvant chemotherapy	1.20 (0.452, 3.189)	0.71
Radiotherapy	1.77 (0.761, 4.108)	0.18
Hormonal therapy	0.88 (0.405, 1.923)	0.75

## Discussion

To gain a better understanding of the complex network of factors affecting tumor behavior and survival outcomes in Indian breast cancer patients, we analyzed demographic characteristics, tumor profiles, and other risk factors, focusing on their associations and also with survival. In our study, breast cancer was more prevalent among postmenopausal women and infrequently occurred in parous women. The frequency of ER+/PR- cases observed in our cohort was consistent with previous research, aligning with a multicentric real-world study that reported 64.1% of patients as ER-positive, 42.2% as PR-negative, and up to 75% as HER2/neu-negative, making hormone receptor-positive/HER2/neu-negative the most common receptor subgroup [13].

Our findings indicate that HER2/neu-positive tumors were more common among older patients, although younger patients also demonstrated a significant predisposition to this receptor profile [14]. Additionally, younger, premenopausal women presented more frequently with high-grade tumors that were strongly correlated with high Ki67 scores, a marker linked to poorer overall survival and an increased need for chemoradiotherapy. Our evaluation of the Ki67 score to gauge tumor aggressiveness parallels findings from Soliman et al., who demonstrated a quantitative association between Ki67 percentages and recurrence and overall survival, reporting notably poor outcomes with Ki67 scores above 15% [15]. Studies have also highlighted the connection between high-grade tumors, elevated Ki67 levels, and reduced overall survival, suggesting that high-grade tumors, due to their greater likelihood of metastasis and resistance to hormone therapy, often require aggressive treatment, including chemotherapy and radiotherapy [16]. This study is one of the very limited studies that presented associations of Ki67 score with clinicopathological parameters such as triple-negative and HER2/neu expressions in more than 200 breast cancer patients in India.

HER2/neu-positive patients in our study often co-expressed hormone receptors (ER and PR), leading to treatment with both hormonal therapy and chemotherapy. Co-expression of HER2/neu and hormone receptors influences tumor aggressiveness and response to treatment. Konecny et al. illustrated a quantitative relationship between HER2/neu and hormone receptors in breast cancer cell lines, explaining the relative resistance of HER2/neu-positive, HR-positive tumors to hormone therapy [16]. Some studies have reported limited clinical benefit from adding hormonal therapy to chemotherapy and HER2-targeted therapy (pre- or postoperative) in HR-positive/HER2-positive patients, irrespective of the crosstalk between hormone receptor and HER2/neu [17]. On the other hand, others have reported significant improvement in overall and disease-free survival with hormonal therapy in combination with chemotherapy and trastuzumab compared to those patients who received only chemotherapy and trastuzumab [18].

Our study also found that aggressive tumor behavior was associated with HER2/neu positivity, ER/PR negativity, and the frequent need for neoadjuvant and adjuvant chemotherapy, echoing findings from previous studies. Such aggressive behavior is typically observed in luminal B, triple-negative, and HER2-positive tumor subtypes. Neoadjuvant chemotherapy is particularly beneficial in managing aggressive breast cancers with HER2 overexpression, helping to mitigate aggressive behavior and improve patient outcomes [19-21]. We observed that premenopausal breast cancer patients in our cohort were more likely to require radiation therapy. This aligns with the findings by Kocaöz et al., who reported that premenopausal patients undergo radiotherapy more frequently than their postmenopausal counterparts. Postoperative adjuvant radiation is well-documented for reducing local recurrence and improving survival in patients undergoing breast-conserving surgery or those with lymph node involvement post-mastectomy [22].

Although median survival was not reached within the study’s approximate 10-year follow-up period, our findings aligned with a prior study on Indian patients, where five- and 10-year overall survival rates were 79% and 66%, respectively, over a 15-year follow-up after primary treatment [23]. Survival HRs were highest among nulliparous women, patients with high-grade tumors, and those with triple-negative status. This subset of patients typically required both neoadjuvant and adjuvant chemotherapy in addition to postoperative radiotherapy, presenting with a higher incidence of adverse events despite extensive treatment. Studies using transcriptomic and genomic analyses have shown that triple-negative breast cancers, owing to their diverse subtypes and unique genetic profiles, exhibit varied clinical behaviors, prognoses, and treatment responses [24]. Additionally, triple-negative tumors tend to have more aggressive biological behavior, with high early-recurrence rates and poorer survival outcomes [25].

A limitation of our study was the relatively small sample size, which led to results that were not statistically significant. Nonetheless, this study is one of the very limited studies that presented associations of Ki67 score with clinicopathological parameters such as triple-negative and HER2/neu expressions in more than 200 breast cancer patients in India.

## Conclusions

Our study found that tumor aggressiveness in Indian breast cancer patients is associated with a high Ki67 score, HER2/neu positivity, and an ER/PR-negative status. Age was associated with certain tumor characteristics, with older patients more likely to present with HER2/neu-positive tumors. HER2/neu-positive tumors often expressed hormone receptors (ER and PR), which suggested a potential benefit from combined chemohormonal therapy. In contrast, younger, premenopausal women were more frequently diagnosed with high-grade tumors and exhibited a positive correlation with elevated Ki67 scores. A high Ki67 score was further linked to poor overall survival and increased requirements for chemoradiotherapy. Both age and menopausal status influenced tumor behavior and the need for radiation therapy, with premenopausal patients more commonly requiring this treatment. This study is among the very few that have examined the association between the Ki67 score and tumor characteristics, including triple-negative and HER2/neu expressions, in a cohort of over 200 breast cancer patients in India.

Although median survival was not reached in our cohort, certain groups exhibited higher event rates, including nulliparous women and patients with high-grade or triple-negative tumors. This emphasizes the importance of tailoring treatment strategies to patient-specific characteristics, such as reproductive history, tumor grade, receptor status, and Ki67 score, to improve patient outcomes.

## Appendices

**Table 8:** Variables with missing data for patients Handling of missing data: This table mentions the number of patients whose data were missing for the study variables. The total number of patients included in the analysis was 389. The number of missing data was minimal for most of the variables except for Ki67. As these are significant clinicopathological characteristics, no assumptions were made, and a data deletion approach was applied during the analysis. Since Ki67 is a crucial biomarker for breast cancer patients, no assumption was made for it either, and the denominator was adjusted accordingly. A noticeable pattern was observed in the variables with missing data, revealing that patients missing data for one variable often lacked information for other variables as well. Additionally, all missing data pertained to biomarkers. The most likely explanation was the unavailability of the biomarker investigation results for these patients.

Variable	Number of patients with missing data
ER.PR receptors co-expression	2
HER2/neu	5
Ki67	145
Triple-negative	5
